# Predictors of non-completion of upper secondary education in Finland based on register data

**DOI:** 10.1177/14034948241257564

**Published:** 2024-08-09

**Authors:** Susanna Raisamo, Tytti Pasanen, Petri Hilli, Timo Ståhl

**Affiliations:** 1 Knowledge base for health and welfare management/Finnish Institute for Health and Welfare (THL), Helsinki, Finland; 2 QSA Quantitative Solvency Analysts Ltd, Helsinki, Finland

**Keywords:** Predictors, register data, school non-completion, upper secondary school

## Abstract

**Aims:**

School non-completion is a public health and educational concern in most countries. This study sought to identify the strongest predictors of the non-completion of upper secondary education based on register data.

**Methods:**

A cross-validated elastic net regression analysis was used to predict school non-completion in a population of 2696 students in the city of Jyväskylä, Finland. The register data included data from the primary social and healthcare register and the educational register.

**Results:**

The non-completion rate was 13.1% (13.4% for males, 12.8% for females). The non-completion of upper secondary education was best predicted by the following seven features (ordered from strongest to weakest): unauthorized absences (odds ratio (OR) = 2.27), out-of-home placement (OR = 2.23), average grade when leaving lower secondary education (OR = 0.73), an anxiety/depression diagnosis (OR = 1.43), visits to child guidance and family counselling centres (OR = 1.17), family poverty (OR = 1.11) and the grade point average in the 5th Grade (OR = 0.95).

**Conclusions:**

**Register data can be utilized to find the strongest predictors of school non-completion. Predictors support multidisciplinary actions preventing non-completion by providing both early signals to target actions more specifically and indicators for monitoring the impact of preventative actions.**

## Introduction

School non-completion is a public health and educational concern in most countries [1–4]. The non-completion rates differ across countries owing to the differences in education systems and ways (non-) completion is measured [5,6]. Nonetheless, Scandinavian countries seem to have the lowest rates compared with other European countries [2,7,8]. In Finland, for example, in general upper secondary education level, the discontinuation percentage is 4 [7]. Finland has nine years of comprehensive education, beginning the year when the child turns seven, and ends when children are 16 years old (in the 9th grade). In 2021, free compulsory education was extended to the age of 18 [8].

School non-completion has been shown to be associated, for example, with a lower socioeconomic status, poorer school performance, poorer health, substance abuse, out-of-home placements during childhood, and mental health problems [4,9–16]. Although the phenomenon along with its risk factors is reasonably well investigated and understood at the individual, family, student and school levels [16], there are still gaps to be filled to further advance our understanding, and to better enable the targeting of activities more specifically to keep students in schools. For example, there is little research on the role of welfare system level factors, such as the use of social and healthcare services in predicting school non-completion [17]. Investigating this issue is relevant at least in the Finnish context, where universal primary health and social service provision for families and children are crucial both for recognizing and preventing child welfare concerns and problems [18].

The purpose of this study is to identify the strongest predictors of non-completion of upper secondary education based on register data with a wide set of potential predictors. Using a machine learning approach enabled us to construct prediction models for school non-completion with the most accurate predictor subsets and, thus, to expand the previous literature [19,20].

## Methods

### Study population and data sources

The data for the present study included students from upper secondary institutions in the city of Jyväskylä, Finland. The data included students whose information from lower secondary school was available, who had started their studies between 2013 (1 January) and 2015 (31 December) and had either graduated (*n*=2344) or not completed their studies (*n*=352) by 2019 (3 August). This resulted in a final sample of 2696 adolescents.

The data on the educational records was drawn from the educational registry of Jyväskylä (from the school administration system Primus), and the health and social data were based on records from the Jyväskylä primary social and healthcare register (the Effica system).

This study was undertaken as part of the wider Finnish Youth Social Impact Bond Program [21] to provide evidence-based support for the city of Jyväskylä to set targets for tackling school non-completion. The Ethics Committee of the Finnish Institute for Health and Welfare (THL) granted approval for the study. The register data was accessed after receiving permission from Hospital Nova in Central Finland (granted November 2021), as well as Jyväskylä Educational Consortium Gradia (granted June 2021), and the City of Jyväskylä’s social and health services (granted June 2021), and the cultural, education and sports services (granted June 2021). The data was anonymized before handing over to the researchers.

### Variables

For the purposes of our study, the non-completion of upper secondary education was defined as an adolescent not graduated from upper secondary education, and not continuing their studies at follow-up in August 2019. Students who were enrolled at an educational institution in 2019 but had not graduated were considered to be continuing their studies and were hence excluded from the analyses. This information was based on the educational records drawn from the educational registry.

Initially, a diverse set of potential predictors was selected to predict the non-completion of upper secondary education. The main criteria for choosing each was a plausible association with school non-completion and availability in registers in the study area and other municipalities, which would enable wider application of the results. For descriptive purposes, the predictors were grouped into nine distinct categories, based on their content and the registry they were obtained from: 1) demographics; 2) school absenteeism; 3) school success; 4) learning support; 5) visits to child guidance and family counselling centres; 6) social welfare measures; 7) health conditions; 8) school behaviour; 9) other. Table I offers a complete list of the predictor variables included in this study, as well as their definitions and classifications.

**Table I.** Descriptive overview of the included registry-based variables used to predict upper secondary school non-completion.

Predictor variables	Description/classification
*Demographics*	
Male	A binary gender classification (male/female).
*School absenteeism*	*Variables indicating different types of absence.*
Authorized absence	Absence justified by the school.
Unauthorized absence	Absence not justified by the school.
Lateness	Arriving late (after the expected time) to class.
Holiday	Absence due to holiday.
Sick	Absence due to illness.
Ascertained absence	Reason for absence is ascertained (e.g. from parents).
Pre-arranged absence	Planned and pre-arranged reason to be elsewhere.
Unknown	Reason for absence is unknown.
Other	Other leaves of absence.
School absenteeism – high	Based on an aggregated binary variable indicating a high number of unauthorized absences, cut-off point being more than seven unauthorized absences during school year.
School absenteeism – some	Based on an aggregated binary variable indicating some (1–7) unauthorized absences during school year.
*School success*	
Grade point average	Numerical calculation, grading scale ranging from 4 (fail) to 10 (excellent) in the general upper secondary education final certificate from 5th to 9th grades.
*Learning support*	*Based on a three category (no support/intensified support/special support)*
Intensified support	Having received intensified and personalized educational support for learning in school by teachers.
Special support	Having received continuous and systematic support (e.g. social and/or mental) for learning in school.
*Visits to child guidance and family counselling centres*	*Based on a binary variable (yes/no) and on the number of visits*
Child guidance and family counselling centre visits	Based on visits (yes/no) to family counselling centres, which provides discussion support for free in matters related to family relations and raising children.
Number of visits	Based on the number of visits to family counselling centres.
*Social welfare measures*	*Based on the decisions made in the child protection system (none/one or more)*
Substitute care	Out-of-home placement decision.
Non-institutional social care in child welfare	Support actions for children and families living in their own homes. Support actions are customized individually according to the needs of families and are settled in non-institutional client plan.
Family poverty	Last-resort social benefit for securing financial means of subsistence when family resources or income are considered insufficient to meet essential living costs.
Other	Other decisions.
*Health conditions*	*Based on the number of doctor visits and F-diagnoses*
Number of doctor visits	Based on the number of doctor visits from 5th grade onwards.
F diagnoses (mental, behavioural and neurodevelopmental disorders)	Based on the diagnoses of doctor’s visit according to the codes of the International Classification of Diseases (ICD 10) (yes/no).
F10	Substance abuse
F20	Schizophrenia
F30	Manic episodes
F40	Phobic anxiety disorders
F50	Eating disorder
F60	Specific personality disorders
F70	Mild intellectual disabilities
F80	Specific developmental disorders of speech and language
F90	Attention-deficit hyperactivity disorders
F99	Mental disorder, not otherwise specified
F4323, F913, F948, F911, F4329, F941, F4324, F6030, F918, F98, F9389, F929, F939, F988, F919, F928, F949, F4325, F912, F989, F910	F diagnoses related to antisocial behaviour, grouping variable (yes/no).
F409, F4320, F413, F4321, F321, F3310, F931, F3130, F3300, F338, F339, F4302, F930, F9380, F4322, F3201, F402, F408, F4300, F439, F323, F438, F341, F4108, F431, F418, F328, F329, F419, F510, F412, F3210, F920, F4109, F411, F3200, F322, F3211	F diagnoses related to depression and/or anxiety, grouping variable (yes/no).
Z004	Psychiatric not otherwise specified diagnosis.
Other	There are codes for some diagnoses, but not one specific for the patient’s condition.
F801, F809, F849, F951, F810, F840, F848, F909, F800, F818, F932, F901, F819, F83, F952, F940, F701, F790, F811, F812, F8411, F8412, F908, F900, F401, F845, F813, F429, F802	F diagnoses related to learning disabilities, grouping variable (yes/no).
F101, F1009, F1000, F121, F1005, F1229, F1220, F125, F129, F1300, F131, F1624, F1852, F199	F diagnoses related to substance abuse, grouping variable (yes/no).
*School behaviour*	*Based on teachers’ marks in school electronic administration system*
Active	Pupil is active in school.
Misbehaviour	Does not conform school norms/practices, including, e.g., detentions.
Neglect studies	Giving insufficient attention to studies.
*Other*	
Switching school	Transferred to another school during the school year.

### Strategy for data analysis

Using a cross-validation elastic net regression analysis, a machine-learning approach, the dataset was randomly split into *k* parts (with *k* = 1000): *k*-1 training datasets to establish the prediction model, and the validation data (*k*th split) that tested the accuracy of the prediction model. In the elastic net regression analysis, variable selection is regulated by the α value, which ranges from 0 to 1 and where lower values generally result in models with more explanatory variables and higher values result in models with fewer explanatory variables (‘features’ in the machine-learning literature). We, therefore, ran the analysis with different levels of the mixing parameter α (0.45, 0.55, 0.65 and 0.75). The predictive power of 53 different variables and their combinations obtained from social and health service registries and education providers were tested. The prediction accuracy was evaluated using an area under the curve (AUC) metric, which is a measure of the accuracy of the discriminatory capacity of classification models. In general, an AUC of 0.5 suggests no discrimination, 0.7 to 0.8 is considered acceptable, 0.8 to 0.9 is considered excellent and more than 0.9 is considered outstanding [22].

Continuous features were scaled using the two-sigma scaling method [23] to ensure comparability with binary features. After the initial runs, the features were narrowed down so that out of the overlapping ones (e.g. regarding grades, school absences and diagnoses for which many indicators were available) the ones showing the greatest predictive power were retained.

Analyses were performed with the R statistical software (version 4.0.4) [24] and the ‘glmnet’ package [25]. Sensitivity models tested different numbers of folds (*k*-value) in the cross-validation (3, 5, 10, 100, and 1500), but the main predictors were not sensitive to these analytical choices.

## Results

### Descriptive results

Of the total study population, 86.9% (*n*=2344) completed their upper secondary education and 13.1% (*n*=352) did not complete it. There were slightly more females (50.6%) than males (49.4%) in the dataset. Non-completion rates, however, were fairly equally distributed between genders (13.4% for males and 12.8% for females). Descriptive information is presented in Table II of the most important features in the present study across the whole dataset and grouped by graduation status.

**Table II.** Descriptive information of the most important features in the study, in the whole dataset and grouped by graduation status. Percentage or mean (SD).

Variable		All		Graduated (*n*=234)		Not completed (*n*=352)	
		*n*	% or mean (SD)	*n*	% or mean (SD)	*n*	% or mean (SD)
Graduated from upper secondary education	Yes	2344	86.9%				
	No	352	13.1%				
Gender	Male	1332	49.4%	1154	86.6%	178	13.4%
	Female	1364	50.6%	1190	87.2%	174	12.8%
Type of upper secondary school	Vocational	1286	47.7%	1067	83.0%	219	17.0%
	Sixth form	1410	52.3%	1277	90.6%	133	9.4%
Intensified support	Yes	118	4.4%	80	67.8%	38	32.2%
	No	2578	95.6%	2264	87.8%	314	12.2%
Special support	Yes	102	3.8%	73	71.6%	29	28.4%
	No	2594	96.2%	2271	87.5%	323	12.5%
Switching school (two-sigma scaled)		2696	0 (0.50)	2344	–0.02 (0.49)	352	0.12 (0.57)
Grade point average (5th grade)		2696	8.16 (0.62)	2344	8.22 (0.59)	352	7.74 (0.63)
Grade point average (9th grade)		2696	7.92 (0.89)	2344	8.03 (0.84)	352	7.18 (0.87)
Family poverty	Yes	363	13.5%	244	67.2%	119	32.8%
	No	2333	86.5%	2100	90.0%	233	10.0%
Non-institutional social care in child welfare	Yes	600	22.3%	419	69.8%	181	30.2%
	No	2096	77.7%	1925	91.8%	171	8.2%
Social welfare measures	Yes	317	11.8%	224	70.7%	93	29.3%
	No	2379	88.2%	2120	89.1%	259	10.9%
No. of doctor visits		2344	12.95 (27.33)	2344	11.48 (23.97)	352	22.69 (42.33)
F diagnoses related to antisocial behaviour	Yes	82	3.0%	48	58.5%	34	41.5%
	No	2614	97.0%	2296	87.8%	318	12.2%
F diagnoses related to depression and/or anxiety	Yes	177	6.6%	105	59.3%	72	40.7%
	No	2519	93.4%	2239	88.9%	280	11.1%
Other diagnosis	Yes	63	2.3%	48	76.2%	15	23.8%
	No	2633	97.7%	2296	87.2%	337	12.8%
F diagnoses related to learning disabilities	Yes	75	2.8%	46	61.3%	29	38.7%
	No	2621	97.2%	2298	87.7%	323	12.3%
Substance abuse diagnosis	Yes	22	0.8%	9	40.9%	13	59.1%
	No	2674	99.2%	2335	87.3%	339	12.7%
Unauthorized school absenteeism – high	Yes	513	19.0%	320	62.4%	193	37.6%
	No	2183	81.0%	2024	92.7%	159	7.3%
Child guidance and family counselling centre visits	Yes	74	2.7%	55	74.3%	19	25.7%
	No	2622	97.3%	2289	87.3%	333	12.7%
Substitute care	Yes	112	4.2%	45	40.2%	67	59.8%
	No	2584	95.8%	2299	89.0%	285	11.0%

**Figure 1.**
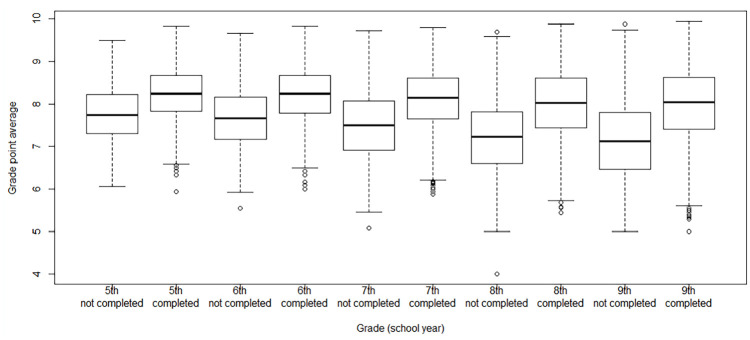
Average grades according to upper secondary school graduation.

Figure 1 shows the distributions of grade point from the 5th grade to the 9th grade among those who completed upper secondary school and among those who did not. This was done because the grade point average was a continuous variable, whereas all the other variables were binary and hence the figure illustrates the results in a more informative way than listing the medians and quartiles for each school year in a table. The final grade point averages in all school years from the 5th until the 9th grade were significantly higher among the students who completed their studies than among those who did not complete them.

### Model fit

The results from the cross-validation were consistent when the number of folds was at least 100, regardless of the level of α. The strongest predictors for school completion were not sensitive to the levels of α. The AUC in models with a reduced set of variables were almost equal or even higher than the AUC of the full dataset (e.g. with α=0.65, the AUC was 0.814 in the reduced dataset and 0.813 in the full dataset) and hence the analysis for the reduced dataset was inspected in more detail.

### Parameters

In the best-predicting models, the non-completion of upper secondary education was predicted by the following seven features ordered from the strongest to the weakest when α=0.65, *k*=1000/1500 with good accuracy (AUC=0.814): unauthorized absences (odds ratio (OR) = 2.27), out-of-home placement (substitute care) (OR = 2.23), average grade when leaving lower secondary education (OR = 0.73), an anxiety or depression diagnosis (OR = 1.43), visits to child guidance and family counselling centre (OR = 1.17), family poverty (OR = 1.11) and average grade in the 5th grade (OR = 0.95). These results were essentially the same with different levels of α (Table III), although the OR estimate for the average grade in the 5th grade reduced as α increased, indicating that this was the weakest predictor for the non-completion of upper secondary education.

**Table III.** Selected features and their estimated odds ratios from the model predicting non-completion of upper secondary education with different alpha levels (*k*=1000).

Feature	α = 0.45		α = 0.55		α = 0.65		α = 0.75	
	Estimate (b)	exp (b)	Estimate (b)	exp (b)	Estimate (b)	exp (b)	Estimate (b)	exp (b)
(Intercept)	0.73	2.08	0.75	2.12	0.56	1.75	0.53	1.70
Grade point average, 5th grade	–0.11	0.90	–0.08	0.92	–0.05	0.95	–0.02	0.98
Grade point average, 9th grade	–0.28	0.76	–0.30	0.74	–0.32	0.73	–0.35	0.70
Family poverty	0.14	1.15	0.13	1.14	0.10	1.11	0.09	1.09
Non-institutional social care in child welfare	0.20	1.22	0.19	1.21	0.16	1.17	0.15	1.16
Depression/anxiety diagnosis	0.39	1.48	0.40	1.49	0.36	1.43	0.37	1.45
Unauthorized school absenteeism – high	0.77	2.16	0.81	2.25	0.82	2.27	0.85	2.34
Substitute care	0.77	2.16	0.80	2.23	0.80	2.23	0.81	2.25
Model AUC	0.815		0.814		0.814		0.814	

AUC: area under the curve; exp (b): the exponentiation of the B coefficient.

**Figure 2.**
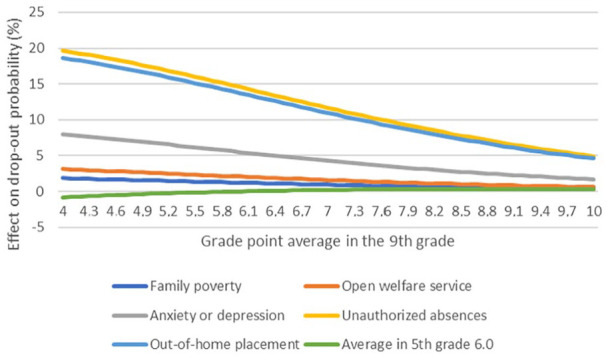
The relative importance of different predictors on school non-completion probability.

These findings are also further illustrated in Figure 2. The figure shows the relative importance of different predictors on the non-completion probability. The grade point is presented on the *x*-axis because it was a continuous variable. For simplicity, the grade point average in the 5^th^ grade was assumed to be 6.0. When the grade point average in the 9th grade was greater than the average in the 5th grade, the non-completion probability decreased.

## Discussion

Based on register data, this study aimed to identify the predictors of the school non-completion of upper secondary education. The results showed that the rate of school non-completion (13%) in a specific Finnish context (city of Jyväskylä) was substantially higher compared with national statistics [7]. It is, thus, reasonable to suggest that a careful identification of the strongest predictors of school non-completion in a local area level may help target preventative actions more specifically. The seven strongest predictors of school non-completion identified in the present context were multifaceted in nature. Below, these predictors are mainly discussed in relation to their strength.

*Unauthorized absence*, which emerged as the strongest predictor of school non-completion, was in line with previous research. A similar finding was reported by Chung and Lee in South Korea [20]. They also used machine-learning algorithms and found that the best predictor for school non-completion was unauthorized absence. Despite the fact that research has consistently shown school absenteeism to be a strong risk factor for non-completion of education [10,16], the focus has predominantly been on the role of school absenteeism without distinguishing different types of absences. It is important to acknowledge that the reasons for absenteeism can be multiple and that authorized absences may also disrupt learning and subsequently have an influence on the non-completion risk [26].

*Out-of-home placement* was the second strongest predictor of adolescent school non-completion. This finding is in line with other studies demonstrating that children who have been placed outside the home are less likely to complete their studies [27]. *Average grade when leaving lower secondary education* was found to be the third strongest predictor of school non-completion. Previous research supports this finding [10,16]. As a novel contribution to the existing literature, we observed its importance as a predictor of school non-completion as early as from the 5th-grade level onwards. Even though *the grade point average in the 5th grade* was the weakest predictor (ranked 7th ) for the non-completion, it could be a potentially important early signal to consider when identifying students at risk of school non-completion.

The fourth strongest predictor of school non-completion in our study was *an anxiety or depression diagnosis*, referring to internalizing mental health disorders. Overall, this finding accords with earlier studies showing that poor mental health is related to school non-completion and that early-onset mental disorders should be considered a key target to reduce non-completion rates [10,13–15]. On the other hand, our result is somewhat in contrast with the earlier literature which has identified that externalizing disorders predict school non-completion better than internalizing disorders [28]. More recent evidence is similar to ours and suggests that internalizing problems have a strong role in predicting school non-completion [29].

Some of the identified predictors of school non-completion in the current study reflect conditions/circumstances that are unreachable by the school. These notable predictors included *visits to child guidance and family counselling centres* (ranked 5th) *and family poverty* (ranked 6th). All of these are signifiers of broader challenges and processes, which may influence the emergence of differences in educational outcomes among children. The pathways leading to these are diverse and too broad to be discussed in detail here. Additionally, the significant effect of family poverty (commonly defined by education and income-based measures) on educational outcomes for children has been widely reported, with a lower socioeconomic family status being linked to poorer educational achievement for children [30]. Based on our findings, visits to child guidance and family counselling centres predicted school non-completion. While these centres serve additional support and guidance roles for adolescents and their families, and thus can be seen as a positive resource in relation to school completion, the reverse finding here may reflect that those who use these services are typically characterized as high-risk students [17].

School non-completion as a phenomenon is complex and influenced by a variety of factors [10–16]. When considering the implications of the current study, the multifaceted nature of the identified predictors suggests that efforts to prevent non-completion are not straightforward. Predictors support multidisciplinary actions preventing non-completion by providing both early signals to target actions more specifically and indicators for monitoring the impact of preventative actions. Furthermore, these findings call for cross-sectoral actions and cooperation between the education, health and social sectors.

### Study strengths and limitations

The strengths of this study were the use of objective register-based data and predictive modelling with machine learning. As the results were based on register information, they were not affected by the self-reported biases that are typically related to survey samples. Using a machine learning approach, we were able to select the most accurate predictor subsets and construct prediction models of school non-completion [19,20]. However, this study is not without limitations. The generalizability of the results outside Finland, particularly in countries with different welfare and educational systems, should be approached with caution. Additionally, there exist potentially relevant factors that may predict school non-completion, but were not available in the register data, such as the students’ ethnicity. Another plausible limitation to acknowledge is that the data was gathered prior to COVID-19 pandemic, which led to disruptions in the education and lives of students. Furthermore, the follow-up time was two years shorter for those students who started their studies in 2015, compared with those who started in 2013, which could have influenced the results because those who had started earlier had more time to complete their education. We assume, however, that this did not cause a major bias because the majority of the students at the upper secondary institutions graduated within the four-year follow-up. Finally, as the data did not cover the whole country but was restricted to one area (city of Jyväskylä), this could represent a limitation. However, there is no clear indication that the main predictors would significantly differ between different cities in Finland.
